# Calcified Chondroid Mesenchymal Neoplasm of the Temporomandibular Joint: A Rare and Recently Described Benign Tumor

**DOI:** 10.1155/crot/8249368

**Published:** 2026-04-27

**Authors:** Jiaxin Yuan, Tyler Lazaro, Laligam N. Sekhar, Kevin C. Lee

**Affiliations:** ^1^ Department of Oral and Maxillofacial Surgery, University of Washington, Seattle, Washington, USA, washington.edu; ^2^ Department of Neurological Surgery, University of Washington, Seattle, Washington, USA, washington.edu

## Abstract

The calcified chondroid mesenchymal neoplasm (CCMN) was first recognized as a distinct pathologic entity in 2021 at the University of Washington, and fewer than 100 cases of CCMN have been described in the literature. The CCMN tumor is characterized by the presence of chondroid matrix elements with an FN1–receptor TK gene fusion. CCMNs frequently arise in the distal extremity joints, but less commonly, they have been known to involve the temporomandibular joint (TMJ). This report details the workup and treatment of a patient with a locally advanced CCMN of the TMJ. Our case highlights the diagnostic challenges, radiographic and histological features, and treatment considerations for TMJ CCMN.

## 1. Introduction

The calcified chondroid mesenchymal neoplasm (CCMN) is a new category of tumor that was first described by Liu et al. in 2021 [1]. CCMN references a spectrum of benign neoplastic lesions characterized by the formation of cartilage or chondroid elements in the presence of an FN1–receptor tyrosine kinase gene fusion [1]. Only a limited number of cases have been reported to date; however, there is a clear predilection for occurrence in the distal extremities with a smaller proportion of cases arising in the temporomandibular joint (TMJ). CCMNs usually occur in middle‐aged adults, but they appear to form sporadically as there are no other known predilections or risk factors [1, 2]. Given the rarity of CCMNs in the head and neck region, head and neck surgeons may lack familiarity with this new entity. It is therefore essential to report and study these cases to better understand their distinctive features, improve diagnostic accuracy, and ensure appropriate treatment for affected patients. In this case report, we present a 61‐year‐old male patient with progressive, painful swelling of the left preauricular region and discuss our management approach.

## 2. Case Presentation

A 61‐year‐old man presented to the University of Washington, Oral and Maxillofacial Surgery Clinic, with a 2‐year history of progressive pain and swelling of the left TMJ region. He endorsed a history of hypertension but was otherwise healthy and had recently immigrated from Kenya. His jaw symptoms started after an assault to the head in 2023. CT imaging at that time revealed a fracture of the left TMJ, though he was unclear about the details of the scan. He did not get surgery at that time, and since then, he has reported progressive and persistent shooting left facial pain with gradual swelling of the left preauricular region. As the swelling worsened, he developed progressive trismus and subjectively endorsed some decreased hearing in the left ear. He denied any visual changes, facial numbness, facial weakness, headaches, otorrhea, or palpable intraoral or cervical masses. On examination, there was a firm, tender swelling of the left preauricular region without overlying skin changes (Figure 1). There was no facial weakness. Maximal interincisal opening was limited to less than 1 cm, though occlusion was midline and not shifted. The otoscopic examination was likewise unremarkable.

FIGURE 1Preoperative clinical images demonstrating (a) left preauricular facial swelling and (b) trismus to less than 1 cm.

**Figure:**
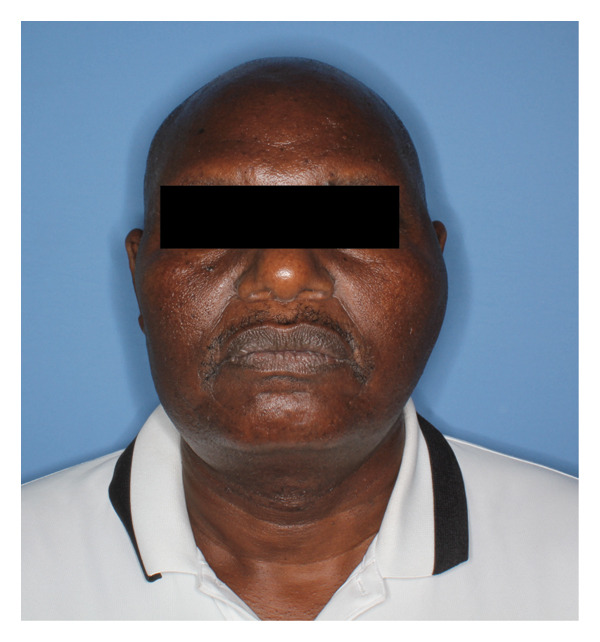
(a)

**Figure:**
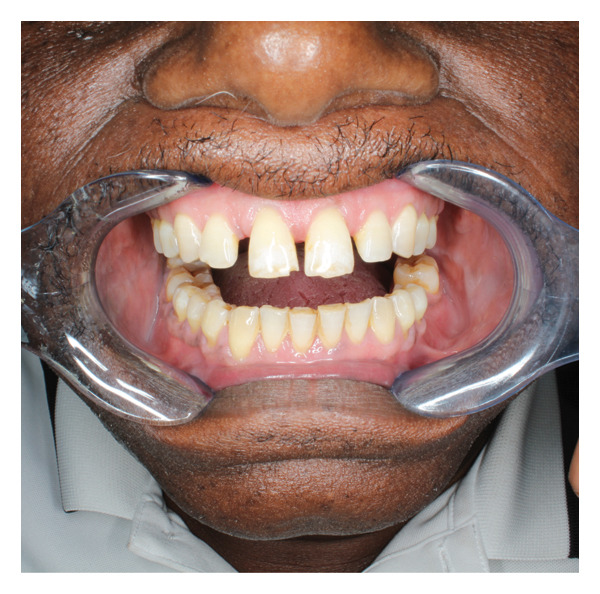
(b)

CT maxillofacial and neck with contrast were obtained and demonstrated a 4.3 × 5.7 × 3.7 cm heterogeneous mass of the left infratemporal fossa (ITF) centered at the left TMJ, with joint space widening, pressure erosion, and remodeling of the left mandibular condyle and ramus (Figure 2). There was extensive erosion of the left lateral skull base with lesion invasion into the middle cranial fossa. The tumor was abutting the internal carotid at the skull base. MRI with contrast was also obtained and similarly demonstrated a heterogeneously enhancing, predominantly T2‐hyperintense mass centered at the left TMJ. The mass displaced but did not invade the main trunk of the facial nerve as it exited the stylomastoid foramen.

FIGURE 2CT maxillofacial with contrast: (a) coronal view soft tissue window demonstrating superior extent of tumor with erosion of the lateral skull base into the middle cranial fossa, (b) coronal view bone window demonstrating skull base invasion with associated pressure erosion and remodeling of the mandibular condyle and ramus, and (c) axial view demonstrating medial extent into the infratemporal fossa.

**Figure:**
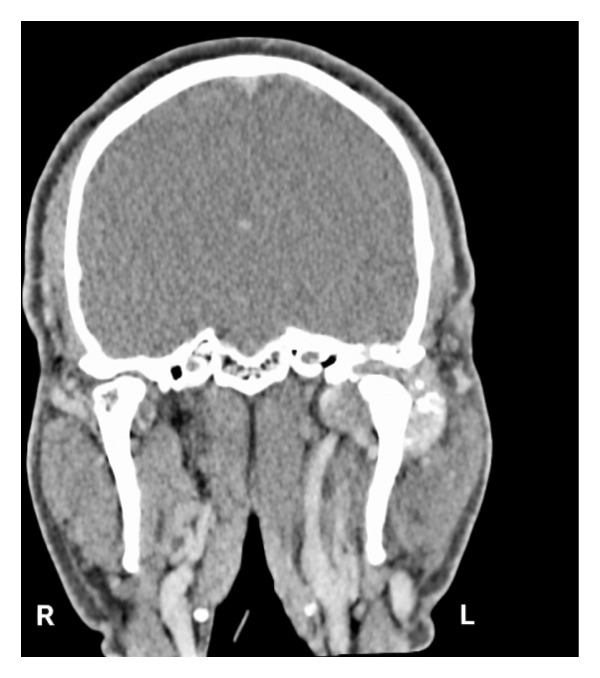
(a)

**Figure:**
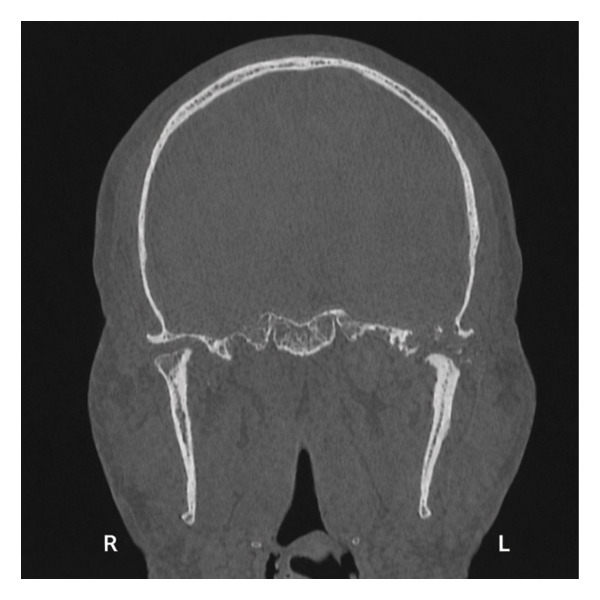
(b)

**Figure:**
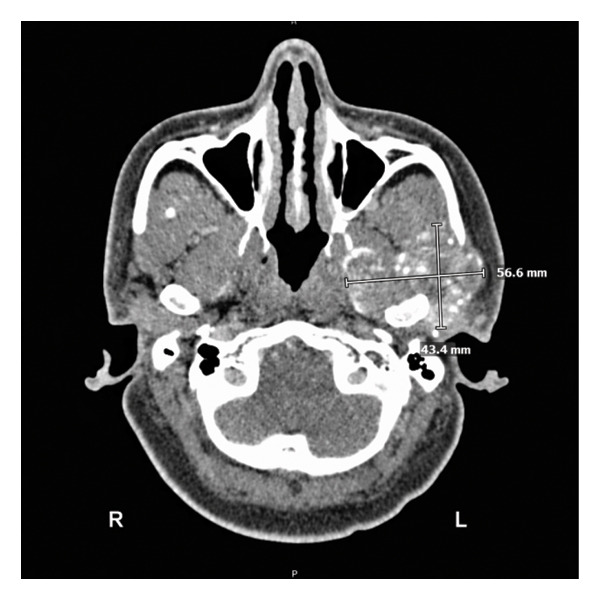
(c)

Given the aggressive features, staging studies were obtained to work up for possible sarcoma or other malignancies. CT neck showed no suspicious cervical lymphadenopathy, CT chest identified a postinflammatory 4‐mm pulmonary nodule, and CT abdomen pelvis did not identify any abnormalities.

The patient was referred for an ultrasound‐guided core needle biopsy. The specimen demonstrated a nodular chondroid neoplasm composed of polygonal to stellate cells with scattered “grungy” and lace‐like (“chicken‐wire”) calcifications. Focal areas showed morphology reminiscent of tenosynovial giant cell tumors (TGCTs). These features were diagnostic of CCMN, with no definitive features of malignancy (Figure 3).

FIGURE 3(a) Low‐power view of the specimen showing a lobular architecture (scanned at 4x magnification using the Aperio AT2), (b) high‐power view showing a classic nodular chondroid neoplasm composed of polygonal to stellate cells with scattered “grungy” and lace‐like (“chicken‐wire”) calcifications (20x magnification), and (c) high‐power view showing focal morphology resembling that of a of tenosynovial giant cell tumor (20x magnification).

**Figure:**
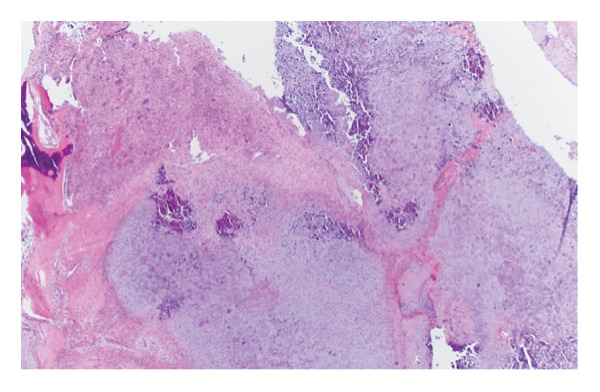
(a)

**Figure:**
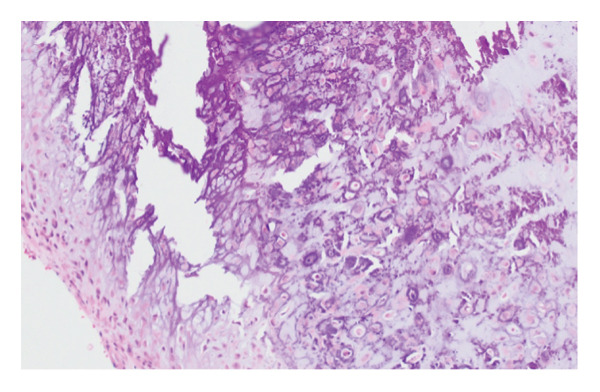
(b)

**Figure:**
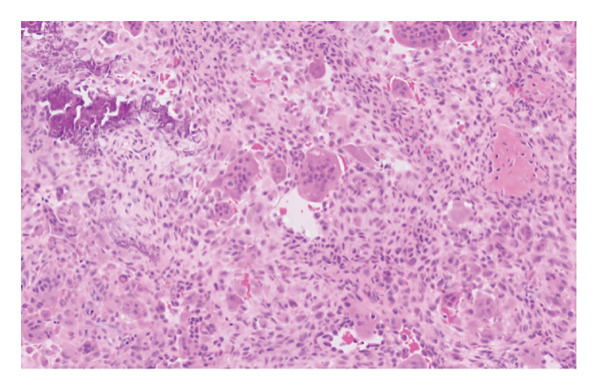
(c)

**FIGURE 5:**
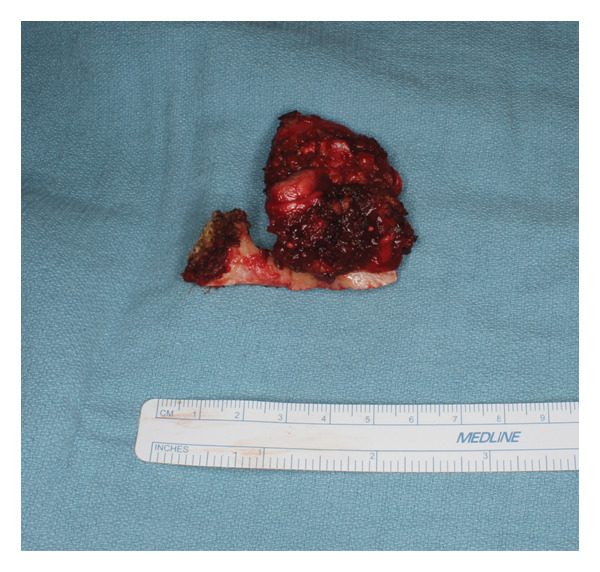
Surgical specimen with tumor encasing the condylar portion.

Given adequate sampling of the tumor and radiographic and pathologic evidence consistent with CCMN, the patient was planned for an ITF compartment resection of the mass with oral/maxillofacial surgery and neurosurgery teams. Three days before the planned surgery, the patient underwent embolization of the left internal maxillary artery to minimize the risk of intraoperative bleeding, which is our standard protocol for open TMJ resection. A nerve integrity monitor (NIM) was used for facial nerve monitoring during the procedure, which is our standard protocol for parotid surgery. A hemicoronal approach with a modified Blair extension was used for exposure (Figure 4). The skin flap was raised, and the zygomatic arch was osteotomized to further increase the medial ITF exposure. Inferiorly, the main trunk of the facial nerve was identified, and the tumor was peeled off the deep surface of the upper division of the facial nerve. A mandibulectomy was made including the coronoid and condyle in the specimen. The neurosurgery team elevated the temporalis muscle for subsequent lateral skull base reconstruction and performed a temporal craniotomy to expose and free the intracranial extradural portion of the tumor superiorly without violating the dura. Medially, the tumor was abutting the petrous carotid canal without bone invasion. The greater superficial petrosal, mandibular, and maxillary nerves were identified and preserved, and there was good NIM signal in all branches on the proximal stimulation of the facial nerve at the labyrinthine segment. With the lateral, inferior, and superior aspects of the tumor freed, the remaining tumor was traced and freed medially off the medial pterygoid. We achieved a gross total resection. The tumor was ill‐defined and composed of multiple cartilaginous loose bodies connected in a fibrous tissue network that made a true en bloc resection impossible (Figure 5).

FIGURE 4(a) Hemicoronal, modified Blair access to approach a high infratemporal fossa tumor, and (b) surgical defect following tumor resection with the zygomatic arch removed for access.

**Figure:**
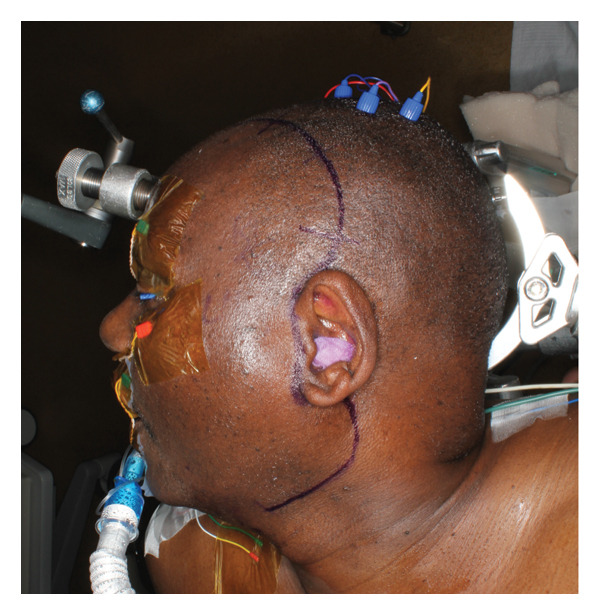
(a)

**Figure:**
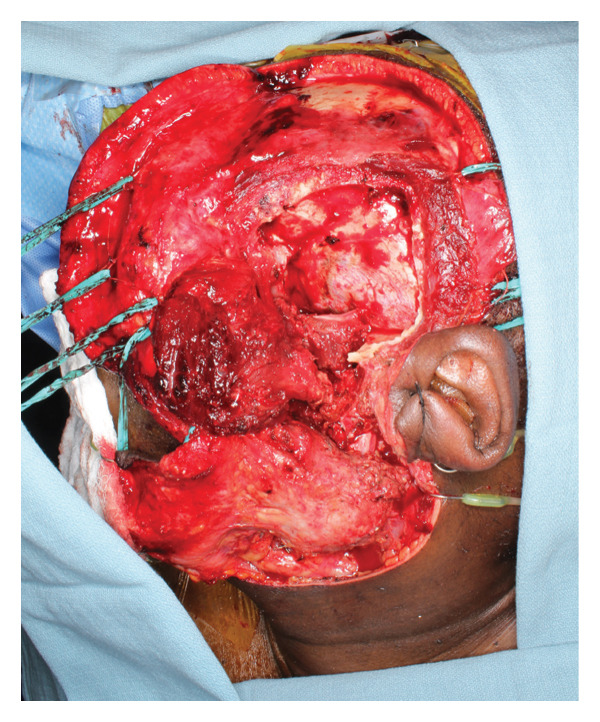
(b)

The skull base was reconstructed with a temporalis muscle flap, and the zygomatic arch was replaced. The mandibular condyle defect was reconstructed with a right costochondral graft. A free abdominal fat graft was used to obliterate dead space around the neocondyle and restore volume (Figure 6). Arch bars were placed, and the patient was placed in maxillomandibular fixation.

FIGURE 6(a) Surgical defect with a temporalis flap rotated to cover the lateral skull base defect and the zygomatic arch replaced, (b) costochondral graft fixated to the mandible with a space between the neocondyle and the lateral skull base, and (c) immediate postoperative CT scan showing ramus–condyle reconstruction.

**Figure:**
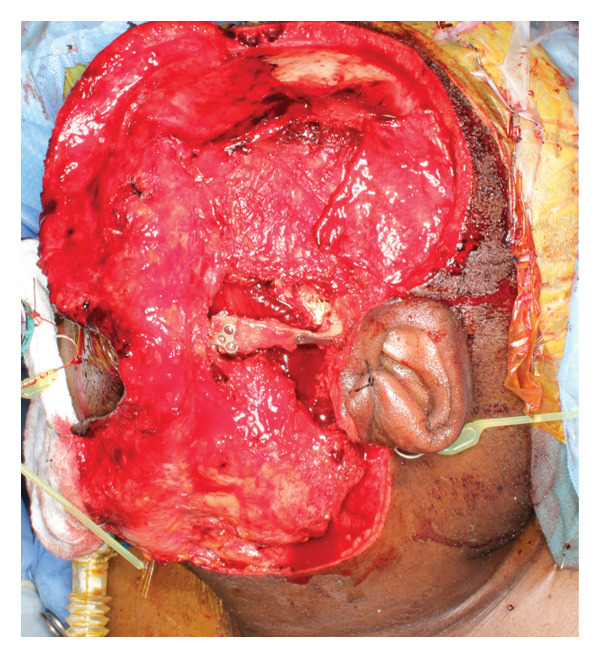
(a)

**Figure:**
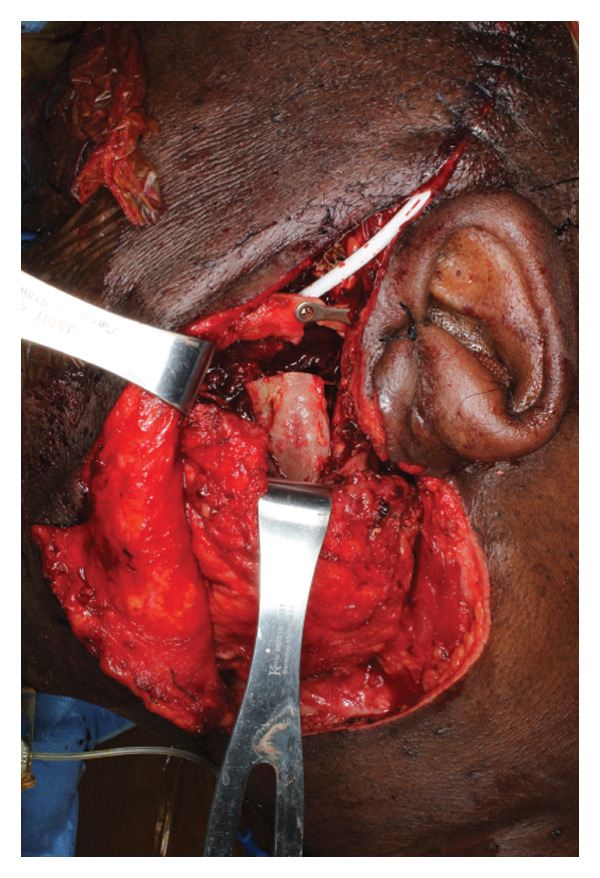
(b)

**Figure:**
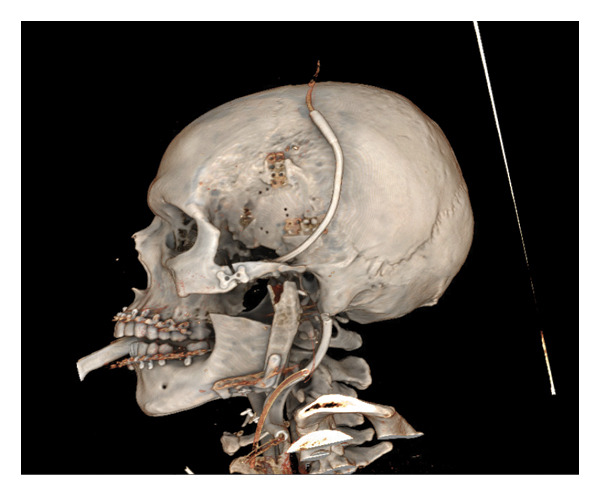
(c)

The patient spent 4 days in the hospital for postoperative monitoring, pain control, and training in self‐care and dietary modifications. Maxillomandibular fixation with elastics was maintained for 4 weeks, at which time his elastics were released, and he demonstrated satisfactory healing and stable occlusion (Figure 7). His trismus had improved with surgery, and his interincisal mouth opening was measured and maintained at 35 mm. Examination revealed weakness of the left frontal branch of the facial nerve, but all other branches were intact. By 6 weeks, he reported good masticatory function. Temporal branch weakness persisted but showed gradual improvement, with full recovery anticipated.

FIGURE 7Postoperative (a) lateral view showing a well‐healed surgical scar and (b) frontal view showing intact facial nerve and some left temporal hollowing.

**Figure:**
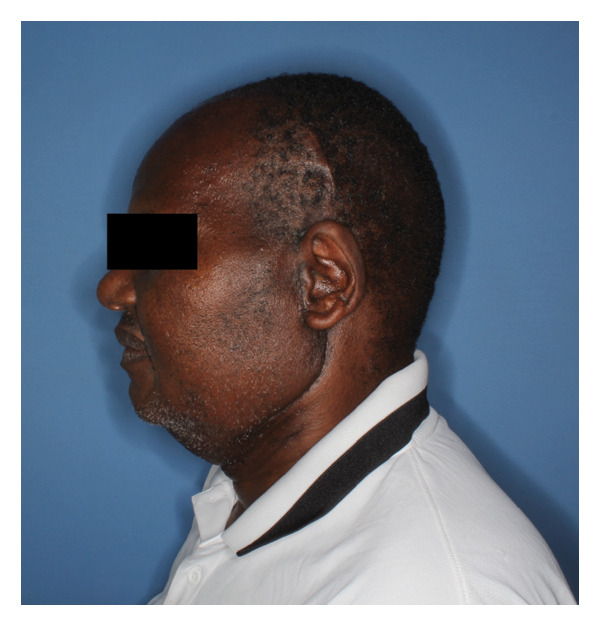
(a)

**Figure:**
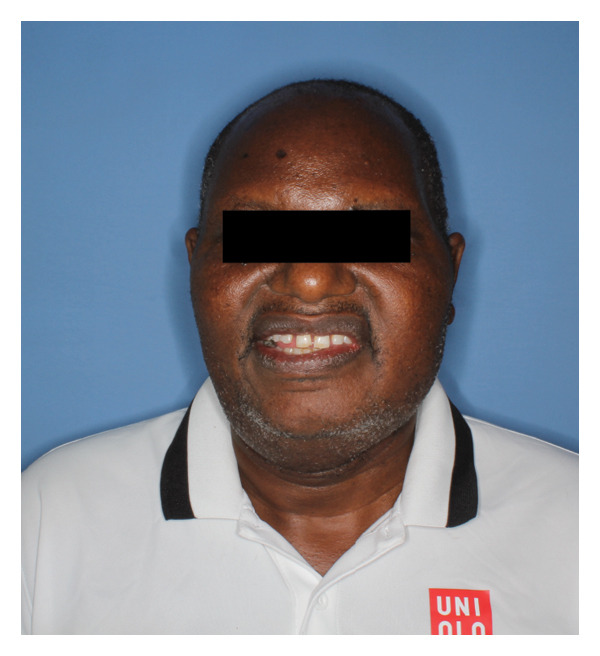
(b)

Final pathology confirmed CCMN and ruled out the presence of a low‐grade sarcoma. The case was presented at the multidisciplinary head and neck tumor board. Given the extensive disease burden, infiltrative growth pattern, and inability to achieve a true en bloc resection, his risk of local recurrence was considered elevated. However, given the benign disease process and lack of evidence supporting adjuvant radiation, the consensus was to proceed with close surveillance. Informed consent was obtained from the patient for publication of the case report.

## 3. Discussion

CCMNs usually present as slow‐growing, soft tissue nodules without systemic symptoms. The radiographic findings are characterized as a well‐defined soft tissue mass containing variable internal calcifications. While CCMNs may cause scalloping of adjacent bone, aggressive findings such as cortical destruction, permeative growth, or periosteal reaction are uncommon, which is consistent with their indolent nature [1–6]. Our case highlights an unusual and more aggressive presentation of this type of tumor.

CCMN lesions exhibit broad morphologic, histologic, and molecular features that encompass entities previously classified as distinct diagnoses. Histologically, these tumors demonstrate a multinodular or lobulated architecture composed of epithelioid to plasmacytoid cells with abundant eosinophilic cytoplasm and eccentric nuclei, arranged in clusters within a variably chondroid and calcified matrix. Osteoclast‐like giant cells and hemosiderin deposition may also be present. Cytologic atypia is generally mild, mitotic activity is rare, and necrosis is absent, supporting an overall low‐grade appearance [1, 3, 4, 7–9].

Despite a relatively consistent cellular component, CCMN exhibits a spectrum of matrix‐associated morphologies. These include chondroma‐like areas with hyaline cartilage–like matrix, chondroblastoma‐like regions with pericellular (“chicken‐wire”) calcification, and calcium pyrophosphate deposition (CPPD)–rich patterns characterized by rhomboid crystal deposition and associated foreign‐body giant cell reaction. These patterns overlap with entities historically described as soft tissue chondroma, chondroblastoma‐like soft tissue tumors, and tophaceous pseudogout, respectively [1–4, 9–11]. A grungy basophilic matrix may also be present and can mimic phosphaturic mesenchymal tumor (PMT), although PMT is a distinct entity and not part of the CCMN spectrum [10].

It is important to recognize that the presence of characteristic histologic features alone is insufficient for the diagnosis of CCMN. Molecular confirmation of recurrent FN1‐related gene fusions, most commonly FN1::FGFR2, is essential (other fusions include FGFR1, TEK, MERTK, and NTRK1, and, less commonly, FGFR3, RET, and PDGFRB) [3, 4, 6–8, 11]. Not all lesions with chondroid or CPPD‐rich morphology harbor these fusions, and CPPD‐rich lesions may also arise in reactive or degenerative conditions. Soft tissue chondroma is a major histologic mimic of CCMN. However, current evidence suggests that only a subset of lesions historically classified as soft tissue chondroma demonstrates FN1‐related fusions, particularly those with chondroblastoma‐like morphology, and should be considered within the CCMN spectrum [1–4, 9–11]. In contrast, conventional soft tissue chondroma remains a distinct benign cartilaginous neoplasm [9]. Accordingly, accurate diagnosis of CCMN requires careful integration of histologic and molecular findings.

In addition to the diagnostic complexity introduced by the reclassification of previously distinct entities into the CCMN spectrum, diagnosis is further complicated by overlap with other cartilaginous tumors that may also show FN1‐related rearrangements. Synovial chondromatosis is a differential for this case since it has a benign but locally aggressive behavior [12]. It can present with multiple calcified intra‐articular nodules and TMJ erosion [13, 14]. Histologically, it is defined by a strikingly nodular proliferation of mature hyaline cartilage with distinct clustering of chondrocytes [14–16]. Although CCMN may exhibit chondroma‐like matrix formation, true hyaline cartilage differentiation is uncommon. S100 and ERG expressions are variable and typically weaker and less diffuse compared with synovial chondromatosis. Importantly, synovial chondromatosis is characterized by distinct gene fusions, most commonly *FN1::ACVR2A*, which are not identified in CCMN [17, 18].

As mentioned previously, PMT can closely mimic CCMN. PMT is a rare neoplasm that can occur in the head and neck and is notable for its association with a paraneoplastic phosphaturic syndrome [19, 20]. PMT can resemble CCMN microscopically, demonstrating a similar grungy basophilic matrix; however, the neoplastic cells are typically oval to spindle‐shaped, in contrast to the plump, plasmacytoid cells characteristic of CCMN. Genetically, more than half of PMTs harbor *FN1* gene fusions, contributing to diagnostic overlap [10, 20]. A key distinguishing feature lies in the clinical presentation, as PMT is associated with FGF‐23–mediated phosphaturic syndrome, leading to hypophosphatemia, muscle weakness, bone pain, and progressive osteomalacia. Another supportive feature is FGF‐23 positivity by CISH or RT‐PCR, which is absent in CCMN [3, 4, 10, 19, 20].

TGCT is a benign lesion that most commonly arises in the distal extremities but can rarely involve the TMJ. Histologically, it is composed of mononuclear cells, multinucleated giant cells, and hemosiderin deposition, including characteristic “ladybird” cells, and most cases harbor CSF1 gene rearrangements [21, 22]. Chondroid TGCT is a rare subtype of TGCT that has been reported to have a predilection for the TMJ and skull base. This subtype often presents with locally aggressive features such as bone destruction like what we saw in our patient. Chondroid TGCT histologically resembles conventional TGCT but contains a nodular chondroid matrix. Importantly, these lesions often harbor FN1‐related fusions rather than CSF1 rearrangements and demonstrate features overlapping with CCMN, including epithelioid cells and a variable chondroid and calcified matrix. Based on these findings, chondroid TGCT is increasingly considered part of the CCMN spectrum rather than a distinct variant of TGCT [1, 2, 4, 9].

Our case is notable for the extensive bony erosion, which raised concern for malignancy. Chondrosarcoma can present with radiographic features overlapping those of CCMN; however, it often demonstrates a characteristic ring‐and‐arc pattern accompanied by permeative endosteal scalloping and cortical destruction, particularly in skull base lesions [23, 24]. On pathology, chondrosarcoma demonstrates infiltrative lobules of hyaline cartilage permeating bone and, unlike CCMN, shows increased cellularity, nuclear atypia, binucleation, and mitotic activity [25]. On molecular testing, chondrosarcomas often harbor IDH1 or IDH2 mutations [26]. Chondroblastic osteosarcoma is another rare malignancy that can arise in the TMJ region. On CT imaging, this osteosarcoma subtype appears as a mixed lytic and sclerotic lesion with cloud‐like or amorphous osteoid mineralization and associated periosteal reaction [27]. In contrast to CCMN, these malignancies typically demonstrate marked cytologic atypia and mitotic activity. In the case of chondroblastic osteosarcoma, malignant osteoid and cartilage‐producing cells are identified on histology [28].

To date, CCMNs are believed to have an indolent clinical course with no evidence of malignant transformation or metastatic potential [1–3]. In the large series reported by Kallen et al., a single case of local recurrence was observed, which the authors attributed to incomplete excision. That patient subsequently underwent re‐excision and remained disease‐free following salvage surgery [3]. Long‐term surveillance after complete excision is recommended to monitor for local recurrence, but no adjuvant therapy is indicated based on current literature and guidelines, given the lack of metastatic potential [2, 3].

In conclusion, CCMN is a recently recognized entity that includes lesions previously considered separate diagnoses. Its rarity, variable matrix, and overlap with other chondroid and calcified soft tissue lesions make diagnosis challenging. Although typically indolent, our case demonstrates an unusual, more aggressive radiographic presentation of CCMN that may mimic a malignant process and should therefore be recognized as a potential diagnostic pitfall. The concept of CCMN as a unifying diagnosis continues to evolve. Accurate classification requires the integration of morphologic, radiographic, and molecular findings to distinguish CCMN from its mimics.

## Funding

No funding was received for this manuscript.

## Conflicts of Interest

The authors declare no conflicts of interest.

## Data Availability

Data sharing is not applicable to this article as no datasets were generated or analyzed during the current study.

## References

[1] Liu Y. J. , Wang W. , Yeh J. et al., Calcified Chondroid Mesenchymal Neoplasms with FN1-receptor Tyrosine Kinase Gene Fusions Including FGFR2, FGFR1, MERTK, NTRK1, and TEK: a Molecular and Clinicopathologic Analysis, Modern Pathology. (2021) 34, no. 7, 1373–1383, 10.1038/s41379-021-00778-3. 33727696

[2] Kao E. Y. , Chen E. , and Liu Y. J. , Calcified Chondroid Mesenchymal Neoplasms, Surgical Pathology Clinics. (2023) 16, no. 4, 545–561, 10.1016/j.path.2023.07.006. 38278609

[3] Kallen M. E. , Chen E. , Liu Y. J. et al., Calcified Chondroid Mesenchymal Neoplasm: Exploring the Morphologic and Clinical Features of an Emergent Entity with a Series of 33 Cases, The American Journal of Surgical Pathology. (2023) 47, no. 8, 1035–1047, 10.1097/PAS.0000000000002115. 37102574

[4] Benard C. , Le Loarer F. , Gomez-Mascard A. et al., Comprehensive Molecular Characterization of a Large Series of Calcified Chondroid Mesenchymal Neoplasms Widening Their Morphologic Spectrum, The American Journal of Surgical Pathology. (2024) 48, no. 8, 991–1004, 10.1097/PAS.0000000000002260. 39016330

[5] Chi A. C. , Schubert E. , Naik K. et al., Calcified Chondroid Mesenchymal Neoplasm Involving the Temporomandibular Joint Region: Case Report and Literature Review, Oral Surgery, Oral Medicine, Oral Pathology and Oral Radiology. (2024) 137, no. 5, 591–598, 10.1016/j.oooo.2023.12.791. 38616481

[6] Fisher Y. , Lacambra M. D. , Almohsen S. S. et al., Expanding the Spectrum of Tyrosine Kinase Fusions in Calcified Chondroid Mesenchymal Neoplasms: Identification of a Novel PDGFRA::USP8 Gene Fusion, Genes Chromosomes & Cancer. (2024) 63, no. 1, 10.1002/gcc.23197. 37642440

[7] Machado I. , Llombart B. , Llombart-Bosch A. et al., Superficial Acral Calcified Chondroid Mesenchymal Neoplasm: a Case Report, Pathology, Research & Practice, 10.1111/cup.14593.

[8] Georgantzoglou N. , Shen G. , Jour G. , and Linos K. , A Case of FN1-fused Calcified Chondroid Mesenchymal Neoplasm of the Hand with Novel FGFR3 Partner Gene, Genes Chromosomes & Cancer. (2023) 62, no. 4, 237–241, 10.1002/gcc.23115. 36504176

[9] Nishino S. , Mori T. , Umezu H. et al., Nosologic Reappraisal of the Recently Proposed Calcified Chondroid Mesenchymal Neoplasm Concept in a Series of 20 Cases, Modern Pathology. (2025) 38, no. 7, 10.1016/j.modpat.2025.100762. 40139500

[10] Gross J. M. and Fritchie K. J. , Calcified Chondroid Mesenchymal Neoplasm, Seminars in Diagnostic Pathology. (2026) 43, no. 1, 10.1016/j.semdp.2025.150978. 41412024

[11] Feng X. , Wang S. , Wei J. et al., Calcified Chondroid Mesenchymal Neoplasm: a Clinicopathological and Molecular Analysis, Journal of Clinical Pathology. (2025) 2024-209806, 10.1136/jcp-2024-209806. 39798957

[12] Liu X. , Huang Z. , Zhu W. , Liang P. , and Tao Q. , Clinical and Imaging Findings of Temporomandibular Joint Synovial Chondromatosis: an Analysis of 10 Cases and Literature Review, Journal of Oral and Maxillofacial Surgery. (2016) 74, no. 11, 2159–2168, 10.1016/j.joms.2016.04.029, 2-s2.0-84994462031. 27238571

[13] Jang B. G. , Huh K. H. , Yeom H. G. et al., Differentiation Between Chondrosarcoma and Synovial Chondromatosis of the Temporomandibular Joint Using CT and MR Imaging, AJNR American Journal of Neuroradiology. (2023) 44, no. 10, 1176–1183, 10.3174/ajnr.A7980. 37652584 PMC10549951

[14] Murphey M. D. , Vidal J. A. , Fanburg-Smith J. C. , and Gajewski D. A. , Imaging of Synovial Chondromatosis with radiologic-pathologic Correlation, RadioGraphics. (2007) 27, no. 5, 1465–1488, 10.1148/rg.275075116, 2-s2.0-34948909216. 17848703

[15] Sink J. , Bell B. , and Mesa H. , Synovial Chondromatosis of the Temporomandibular Joint: Clinical, Cytologic, Histologic, Radiologic, Therapeutic Aspects, and Differential Diagnosis of an Uncommon Lesion, Oral Surgery, Oral Medicine, Oral Pathology and Oral Radiology. (2014) 117, no. 3, e269–e274, 10.1016/j.oooo.2013.04.020, 2-s2.0-84893922342. 23850367

[16] Moraes R. M. , Lescura C. M. , Amstalden E. M. I. , and Anbinder A. L. , Temporomandibular Joint Synovial Chondromatosis with a Giant Cell Component, International Journal of Oral and Maxillofacial Surgery. (2025) 54, no. 25, 01290–01291, 10.1016/j.ijom.2025.05.003. 40436718

[17] Amary F. , Perez-Casanova L. , Ye H. et al., Synovial Chondromatosis and Soft Tissue Chondroma: Extraosseous Cartilaginous Tumor Defined by FN1 Gene Rearrangement, Modern Pathology. (2019) 32, no. 12, 1762–1771, 10.1038/s41379-019-0315-8, 2-s2.0-85068548217. 31273315 PMC6882679

[18] Agaram N. P. , Zhang L. , Dickson B. C. et al., A Molecular Study of Synovial Chondromatosis, Genes Chromosomes & Cancer. (2020) 59, no. 3, 144–151, 10.1002/gcc.22812. 31589790 PMC7147082

[19] Lee J. C. , Su S. Y. , Changou C. A. et al., Characterization of FN1–FGFR1 and Novel FN1–FGF1 Fusion Genes in a Large Series of Phosphaturic Mesenchymal Tumors, Modern Pathology. (2016) 29, no. 11, 1335–1346, 10.1038/modpathol.2016.137, 2-s2.0-84979256639. 27443518 PMC13417894

[20] Qari H. , Hamao-Sakamoto A. , Fuselier C. , Cheng Y. S. , Kessler H. , and Wright J. , Phosphaturic Mesenchymal Tumor: 2 New Oral Cases and Review of 53 Cases in the Head and Neck, Head and Neck Pathology. (2016) 10, no. 2, 192–200, 10.1007/s12105-015-0668-3, 2-s2.0-84947447612. 26577211 PMC4838976

[21] Xu H. , Li Y. , Lin N. , Ye Z. , and Niu X. , Tenosynovial Giant Cell Tumor: Mechanisms and Advances in Targeted Treatments, Critical Reviews in Oncology. (2025) 216, 10.1016/j.critrevonc.2025.104951. 40953758

[22] Assi T. , Moussa T. , Ngo C. et al., Therapeutic Advances in Tenosynovial Giant Cell Tumor: Targeting the CSF1/CSF1R Axis, Cancer Treatment Reviews. (2025) 134, 10.1016/j.ctrv.2025.102904. 40020639

[23] Murphey M. D. , Walker E. A. , Wilson A. J. , Kransdorf M. J. , Temple H. T. , and Gannon F. H. , Imaging of Primary Chondrosarcoma: Radiologic-Pathologic Correlation, RadioGraphics. (2003) 23, no. 5, 1245–1278, 10.1148/rg.235035134. 12975513

[24] Hasegawa H. , Shin M. , Niwa R. et al., Revisitation of Imaging Features of Skull Base Chondrosarcoma in Comparison to Chordoma, Journal of Neuro-Oncology. (2022) 159, no. 3, 581–590, 10.1007/s11060-022-04097-2. 35882753

[25] Limaiem F. , Davis D. D. , and Sticco K. L. , Chondrosarcoma, StatPearls [Internet]. Treasure Island, FL, 2025, StatPearls Publishing. 30844159

[26] Meijer D. M. , Venneker S. , Ameline B. et al., An Integrated Clinical Genomic and Transcriptomic Subgrouping of Central Chondrosarcoma, Modern Pathology. (2025) 38, no. 12, 10.1016/j.modpat.2025.100894. 40976495

[27] Kawaguchi Y. , Matsumoto S. , Manabe J. , Matsushita Y. , Okazaki H. , and Nishida J. , Different and Identical Features of Chondroblastic Osteosarcoma and Chondrosarcoma: Highlights on Radiography and Magnetic Resonance Imaging, Skeletal Radiology. (2009) 38, no. 6, 531–539, 10.1007/s00256-008-0645-5. 19251535

[28] Who Classification of Tumours Editorial Board , Soft Tissue and Bone Tumours, 2020, 5th edition, International Agency for Research on Cancer, Lyon, France.

